# Supplementary material for: A spatial simulation approach to account for protein structure when identifying non-random somatic mutations
Source: BMC Bioinformatics. 2014 Jul 3;15:231. doi: 10.1186/1471-2105-15-231 (PMC4227039; doi:10.1186/1471-2105-15-231)
Supplement: Additional file 1 — Cosmic query. Shows the SQL query used to extract mutations from the COSMIC database. [file 1471-2105-15-231-S1.docx]

Below is the SQL query used to generate the raw data from Cosmic Database version 65.

drop table test;

create table test

as

SELECT GSO.gene_name,SA.id_sample, upper(REPLACE(sa.sample_name, '-','')) as sample_name,gsm.id_mutation, SM.AA_mut_start, SM.AA_MUT_STOP,length(TR.TRANSCRIPT_AA_SEQ) as AA_len, GSO.swissprot_accession,

SA.ID_SOURCE_SPEC,SA.ID_source, GSM.ID_MUT_SOMATIC_STATUS, gsm.ID_MUT_VERIF_STATUS, SA.ID_SOURCE_TISSUE_ORIGIN, TR.accession_number, GS.WHOLE_GENE_SCREEN,GS.WHOLE_GENOME_SCREEN

FROM ANALYSED_GENE_SAMPLE T, GENE_STUDY GS, Gene_sample_mutation GSM, sequence_mutation SM ,cosmic_tumour_sample_overview SA, gene_study_transcript GST, transcript TR, Gene_SOM GSO

where GS.id_gene_study = T.id_gene_study and GSM.id_ags=T.ID_AGS and SM.id_mutation=GSM.id_mutation and SA.id_sample=T.id_sample and GST.id_gene_study=GS.id_gene_study and TR.id_transcript = GST.ID_TRANSCRIPT and GSO.id_gene=TR.id_gene

and GSM.ID_MUT_SOMATIC_STATUS in (1, 2, 5) and SM.id_mut_type_AA=22 and (GS.WHOLE_GENE_SCREEN='y' or GS.WHOLE_GENOME_SCREEN='y' or sa.sample_name like 'TCGA%' or sa.sample_name like 'ICGC%' or sa.sample_name like 'CGP%' or sa.sample_name like 'LUAD%' or sa.sample_name like 'LC_%')

order by gs.id_gene,SM.AA_mut_start,sa.sample_name;

delete from test

where (id_sample,id_mutation,aa_mut_start) in (select id_sample,id_mutation,aa_mut_start

from test group by id_sample,id_mutation,aa_mut_start having count(*)>1) and rowid not in (select min(rowid) from test group by id_sample,id_mutation,aa_mut_start having count(*)>1);

select * from test;

A visual representation of the table relationships within the database:

ID_TRANSCRIPT

ID_GENE

TRANSCRIPT_AA_SEQ

....

TRANSCRIPT

ID_GENE

GENE_NAME

CHROMOSOME

GENE_SOM

ID_GENE_STUDY

ID_GENE

ID_GENE_FUSION

ID_GENE_STUDY_TYPE

ID_PAPER

GENE_STUDY

ID_AGS

ID_GENE_STUDY

ID_SAMPLE

LOH

PAPER_SAMPLE_NUMBER

ANALYSED_GENE_SAMPLE

ID_AGS

ID_MUTATION

PERCENT_MUT_ALLELE

ID_MUT_SOMATIC_STATUS

GENE_SAMPLE_MUTATION

ID_SAMPLE

ID_TUMOUR

ID_SOURCE

IS_CGP

ID_IND....

COSMIC_TUMOUR_SAMPLE_OVERVIEW

ID_GENE_STUDY

ID_TRANSCRIPT

GENE_STUDY_TRANSCRIPT

ID_MUTATION

ID_TRANSCRIPT

ID_MUT_TYPE

ID_MUT_TYPE_AA

...

SEQUENCE_MUTATION

Restrictions imposed upon the mutation accepted for the study.

1) COSMIC studies that come to sequencing the whole gene or the whole genome, or that are from CGP (Cancer Genome Project), TCGA (The Cancer Genome Atlas), ICGC (International Cancer Genome Consortium), LUAD (Lung Adenocarcinoma), LC (Lung Cancer): gene_study.whole_gene_screen = 'y' or

gene_study.whole_genome_screen='y' or

cosmic_tumour_sample_overview.sample_name like 'TCGA%' or

cosmic_tumour_sample_overview.sample_name like 'ICGC%' or

cosmic_tumour_sample_overview.sample_name like 'CGP%' or sa.sample_name like 'LUAD%' or

cosmic_tumour_sample_overview.sample_name like 'LC_%'

2) Variants that are somatic: GSM.ID_MUT_SOMATIC_STATUS in (1, 2). Dictionary of mutation status is shown in table 1.

Table 1. Mutation status dictionary in mut_status_dic.

| 1 | Confirmed somatic variant | somatic_status |
| --- | --- | --- |
| 2 | Reported in another cancer sample as somatic | somatic_status |
| 3 | Confirmed germline variant | somatic_status |
| 21 | Likely cancer causing | consequence |
| 22 | Possible cancer causing | consequence |
| 23 | Unknown consequence | consequence |
| 0 | Not specified | somatic_status |
| 4 | Reported in another sample as germline | somatic_status |
| 5 | Variant of unknown origin | somatic_status |
| 6 | Not curated | somatic_status |
| 25 | To be decided | consequence |

3) Mutation type:

Substitution – missense: SM.id_mut_type_AA=22

Table 2. Part of mutation type dictionary in mut_type_dic

| 11 | Insertion |
| --- | --- |
| 12 | Deletion |
| 13 | Complex |
| 32 | Nonstop extension |
| 21 | Substitution - Nonsense |
| 22 | Substitution - Missense |
| 23 | Substitution - coding silent |
| 24 | Insertion - In frame |
| 25 | Insertion - Frameshift |

4) Delete the duplicated rows that have the same gene name, sample number and mutation positions.
